# SARS-CoV-2 Seroprevalence in Household Domestic Ferrets (*Mustela putorius furo*)

**DOI:** 10.3390/ani11030667

**Published:** 2021-03-02

**Authors:** Jacobo Giner, Sergio Villanueva-Saz, Ana Pilar Tobajas, María Dolores Pérez, Ana González, Maite Verde, Andrés Yzuel, Ana García-García, Víctor Taleb, Erandi Lira-Navarrete, Ramón Hurtado-Guerrero, Julián Pardo, Llipsy Santiago, José Ramón Paño, Héctor Ruíz, Delia Lacasta, Antonio Fernández

**Affiliations:** 1 Menescalia Veterinary Clinic, 46020 Valencia, Spain; jacoboginer@gmail.com; 2 Department of Animal Pathology, Veterinary Faculty, University of Zaragoza, 50009 Zaragoza, Spain; anagi@unizar.es (A.G.); mverde@unizar.es (M.V.); hectorruiz353@gmail.com (H.R.); dlacasta@unizar.es (D.L.); 3 Clinical Immunology Laboratory, Veterinary Faculty, University of Zaragoza, 50009 Zaragoza, Spain; andresyzuel17@gmail.com; 4 Departament of Pharmacology and Physiology, Veterinary Faculty, University of Zaragoza, 50009 Zaragoza, Spain; 5 Instituto Agroalimentario de Aragón-IA2, University of Zaragoza, 50009 Zaragoza, Spain; atobajas@unizar.es (A.P.T.); dperez@unizar.es (M.D.P.); 6 Department of Animal Production and Sciences of the Food, Veterinary Faculty, University of Zaragoza, 50009 Zaragoza, Spain; 7 Institute for Biocomputation and Physics of Complex Systems (BIFI), Mariano Esquillor s/n, Campus Rio Ebro, Edificio I+D, University of Zaragoza, 50009 Zaragoza, Spain; anaauxi.garcia@gmail.com (A.G.-G.); victorzgz1993@hotmail.com (V.T.); erandi@bifi.es (E.L.-N.); rhurtado@bifi.es (R.H.-G.); 8 Aragon I+D Foundation (ARAID), 50018 Zaragoza, Spain; pardojim@unizar.es; 9 Laboratorio de Microscopías Avanzada (LMA), Mariano Esquillor s/n, Campus Rio Ebro, Edificio I+D, 50018 Zaragoza, Spain; 10 Department of Cellular and Molecular Medicine, School of Dentistry, University of Copenhagen, 1165 Copenhagen, Denmark; 11 Aragon Health Research Institute (IIS Aragón), 50009 Zaragoza, Spain; llipsysg@gmail.com; 12 Department of Microbiology, Pediatrics, Radiology and Public Health, University of Zaragoza, 50009 Zaragoza, Spain; 13 Infectious Disease Department, University Hospital Lozano Blesa, 50009 Zaragoza, Spain; joserrapa@gmail.com

**Keywords:** coronavirus disease 2019 (COVID-19), household ferrets, ELISA, SARS-CoV-2, serology

## Abstract

**Simple Summary:**

Animal infections with SARS-CoV-2 have been reported in different countries and several animal species have been proven to be susceptible to infection with SARS-CoV-2 both naturally or by experimental infection. Moreover, infections under natural conditions in more than 20 mink farms have been reported where humans could have been the source of infection for minks. However, little information is available about the susceptibility of pet animals under natural conditions and currently there is no SARS-CoV-2 epidemiological assessment occurrence in household ferrets. In this study, the presence of SARS-CoV-2 antibodies was evaluated in serum samples obtained from 127 household ferrets (*Mustela putorius furo*) in the Province of Valencia (Spain). Two ferrets tested positive to SARS-CoV-2 (1.57%) by in-house enzyme-linked immunosorbent assay based on receptor binding domain (RBD) of Spike antigen. Furthermore, anti-RBD SARS-CoV-2 antibodies persisted at detectable levels in a seropositive SARS-CoV-2 domestic ferret beyond 129 days since the first-time antibodies were detected. This study reports for the first time the evidence of household pet ferrets exposure to SARS-CoV-2 in Spain to date.

**Abstract:**

Animal infections with SARS-CoV-2 have been reported in different countries and several animal species have been proven to be susceptible to infection with SARS-CoV-2 both naturally and by experimental infection. Moreover, infections under natural conditions in more than 20 mink farms have been reported where humans could have been the source of infection for minks. However, little information is available about the susceptibility of pet animals under natural conditions and currently there is no SARS-CoV-2 epidemiological assessment occurrence in household ferrets. In this study, the presence of SARS-CoV-2 antibodies was evaluated in serum samples obtained from 127 household ferrets (*Mustela putorius furo*) in the Province of Valencia (Spain). Two ferrets tested positive to SARS-CoV-2 (1.57%) by in-house enzyme-linked immunosorbent assay based on receptor binding domain (RBD) of Spike antigen. Furthermore, anti-RBD SARS-CoV-2 antibodies persisted at detectable levels in a seropositive SARS-CoV-2 domestic ferret beyond 129 days since the first time antibodies were detected. This study reports for the first time the evidence of household pet ferrets exposure to SARS-CoV-2 in Spain to date.

## 1. Introduction

Coronaviruses infect a wide range of animal species including humans, causing a diverse array of diseases, although each coronavirus tends to be species-specific. Coronaviruses are subdivided into four genera: alpha, beta, gamma and delta-coronaviruses, based on phylogenetic clustering [1]. Alphacoronavirus and beta-coronavirus are commonly associated with respiratory illness in humans and gastroenteritis in animals. Severe acute respiratory syndrome coronavirus 2 (SARS-CoV-2), a beta-coronavirus, is the causal agent of Coronavirus Disease 2019 (COVID-19) that was declared as pandemic by the World Health Organization (WHO) in March 2020. SARS-CoV-2 was originated in animals and is now easily and efficiently transmitted among people, causing predominantly respiratory disease with varying degrees of severity.

Two distinct alphacoronaviruses have been described in domestic ferrets: ferret enteric coronavirus (FRECV), causative of epizootic catarrhal enteritis, and ferret systemic coronavirus (FRSCV) which causes a multisystemic infection in ferrets closely resembling the granulomatous or dry form of feline infectious peritonitis (FIP). It is unknown whether FRSCV and FRECV are genetically distinct coronaviruses of ferrets [2] and, currently, there is no obvious mechanistic link between FRECV and FRSCV that is equivalent to the “internal mutation” of feline coronavirus [3]. Equally, it is unknown whether antibodies against FRECV or FRSCV would be able to neutralize SARS-CoV-2 or contribute to further disease in ferrets via antibody-dependent enhancement of infection [3]. Ferrets are presumed to have been domesticated for more than two thousand years and have become more popular as pets in recent decades. This species is also widely used as a small animal model in the study of some human viral infections, such as influenza A virus and SARS coronavirus [1,4]. Laboratory studies also showed that cats and ferrets are highly susceptible to SARS-CoV-2 isolated from humans [5]. In recent experimental investigations of SARS-CoV-2 infections of domestic species, isolation of the virus from upper respiratory samples from a small sample of ferrets was consistent with the infection [6,7]. However, there are no published reports so far on the investigation of SARS-CoV-2 in domestic pet ferrets from households.

The aim of the present study was to determine the seroprevalence of SARS-CoV-2 by detecting anti-SARS-CoV-2 antibodies using the ELISA method from January to November 2020 in household ferrets living in the Province of Valencia, Spain, a geographic area seriously affected by COVID-19. The results obtained by this sero-survey could contribute to broaden the knowledge of the role played by pet ferrets in the context of COVID-19.

## 2. Materials and Methods

### 2.1. Study Area, Sampling and Data Collection

One hundred and ninety-four residual client-owned ferrets serum samples were obtained from 127 patients seen for medical reasons or routine healthcare check-ups at Menescalia Veterinary Center in Valencia, in the Province of Valencia, Spain (39°28′12.864” N, 0°22′36.48” W). Serum samples were collected aseptically by cranial cava venipuncture with the consent of the owner during the period from January to November 2020. Equally, one milliliter of blood was collected and introduced in another tube containing ethylenediaminetetraacetic acid anticoagulant to perform a hematology profile in those patients which needed it for medical reasons.

From the 194 samples obtained, 37 serum samples were collected in the first quarter of 2020, 77 serum samples in the second quarter, 44 serum samples in the third quarter and the last 36 serum samples during October and November. Residual separated serums were stored at −20 °C until were processed.

Three client-owned ferrets testing positive to ferret coronaviruses (FRECV (one animal) and FRSCV (two animals)) were included in this survey to study the possibility of antigenic cross-reactivity between RBD protein of SARS-CoV-2 and RBD protein of FRECV and FRSCV.

Samples from a total of 44 ferrets were obtained at different time points during the study period. Two samples were obtained from 29 ferrets, three samples each from 10 ferrets, four samples each from three ferrets, five samples from one ferret and six samples from one ferret throughout the study. Table 1 shows each patient with its health condition and each SARS-CoV-2 serological results obtained by ELISA.

### 2.2. Expression and Purification of RBD of Spike

The DNA sequence encoding amino acid residues 319–541 (RVQPTESIVRFPNITNLCPFGEVFNATRFASVYAWNRKRISNCVADYSVLYNSASFSTFKCYGVSPTKLNDLCFTNVYADSFVIRGDEVRQIAPGQTGKIADYNYKLPDDFTGCVIAWNSNNLDSKVGGNYNYLYRLFRKSNLKPFERDISTEIYQAGSTPCNGVEGFNCYFPLQSYGFQPTNGVGYQPYRVVVLSFELLHAPATVCGPKKSTNLVKNKCVNF) of the RBD was codon optimized and synthesized by Gen-Script (USA) for expression in HEK293 cells. The DNA, containing, at the 5′-end, a recognition sequence for KpnI, and, at the 3′end, a stop codon and a recognition sequence for XhoI, was cloned into a modified pHLSec containing, after the secretion signal sequence, a 12 × His tag, a super folder GFP and a Tobacco Etch Virus (TEV) cleavage site, rendering the vector pHLSec-12His-GFP-TEV-SRBD. Both the synthesis of the RBD construct and the engineered pHLSec together with the cloning of RBD into pHLSec-12His-GFP-TEV were performed by GenScript. pHLSec-12His-GFP-TEV-RBD was transfected into HEK293F cell line (Thermo Fisher Scientific, Waltham, MA, USA) as described below. Cells were grown in suspension in a humidified 37 °C and 8% CO_2_ incubator with rotation at 125 rpm. Transfection was performed at a cell density of 2.5 × 10^6^ cell/mL in fresh F17 serum-free media with 2% Glutamax and 0.1% P188. For each 150 mL of culture, 450 μg of the plasmid (1μg/μL) was diluted to 135 μL and sterilized 1.5 M NaCl. This mixture was added to each 150 mL cell culture flask and incubated for 5 min in the incubator. Then, 1.35 mg of PEI-MAX (1 mg/mL) was mixed to 135 μL with sterilized 1.5 M NaCl and added to the cell culture flask. Cells were diluted 1:1 with pre-warmed media supplemented with valproic acid 24 h post-transfection to a final concentration of 2.2 mM. Cells were harvested six days post-transfection by spinning down at 300× g for 5 min, after which the supernatants were collected and centrifuged at 4000× g for 15 min. Supernatant was dialyzed against buffer A (25 mM TRIS pH 7.5, 300 mM NaCl) and loaded into a His-Trap Column (GE Healthcare, Chicago, IL, USA). Protein was eluted with an imidazol gradient in buffer A from 10 mM up to 500 mM. Buffer exchange to 25 mM TRIS pH 7.5, 150 mM NaCl (buffer B) was carried out using a HiPrep 26/10 Desalting Column (GE Healthcare). TEV protease was then added in a ratio 1:50 (TEV:SRBD) to the fusion construct in order to cleavage the His-GFP. After 20 h of reaction at 18 °C, the cleavage was satisfactorily verified through SDS-PAGE. TEV protease and GFP were removed from the solution using a His-TrapColumn (GE Healthcare), and the SRBD was collected from the flow-through. Quantification of protein was carried out by absorbance at 280 nm using the theoretical extinction coefficient, ε280 nm(RBD) = 33,350 M^−1^cm^−1.^

### 2.3. Detection of SARS-CoV-2 Antibodies by In-House ELISA

An in-house indirect ELISA for the detection of IgG specific for RBD of Spike was established. Ninety-six–well plates were coated overnight, at 4 °C with 100 ng RBD protein in phosphate buffered saline (PBS). Subsequently, the coating solution was removed and the plate was washed three times with 200 μL per well of PBS+TWEEN 0.05 % (PBST). 300 μL of PBST containing 3% dry skimmed milk was added to each well as blocking solution. Plate was incubated with blocking solution for 1 h at 37 °C in a moist chamber. 100 µL of ferret sera, diluted 1:100 in PBS containing 0.05% Tween 20 and 1% dry skimmed milk (PBST-M), was added to each well. The plates were incubated for 1 h at 37 °C in a moist chamber. After washing the plates for 30 s 6 times with PBST followed by 1 wash with PBS for 1 min, 100 µL/well of horseradish peroxidase multi-species conjugate (Thermo Fisher Scientific, Waltham, MA, USA) diluted 1:100,000 in PBST-M was added per well. The plates were incubated for 1 h at 37 °C in a moist chamber and were washed again with PBST and PBS as described above. The substrate solution (ortho-phenylene-diamine) and stable peroxide substrate buffer (Thermo Fisher Scientific, Waltham, MA, USA) was added at 100 µL per well and developed for 20 ± 5 min at room temperature in the dark. The reaction was stopped by adding 100 µL of 2.5 M H_2_SO_4_ to each well. Absorbance values were read at 492 nm. in an automatic microELISA reader (ELISA Reader Labsystems Multiskan, Midland, ON, Canada). To evaluate the signal of this ELISA when human plasma is assayed, ELISA was performed using human residual plasma from healthy individuals prior to COVID-19 pandemic (*n* = 24) and from SARS-Cov-2 PCR-positive patients (*n* = 99) with a serological result obtained by a commercial quantitative ELISA (SARS-CoV-2 S1RBD IgG ELISA Kit, MyBiosource, San Diego, CA, USA). The correlation between the absorbances obtained in the commercial ELISA test and the in-house ELISA test showed a R square of 0.810 (regression analysis performed with IBM SPSS v.22). As a positive control, each plate included serum from a human patient diagnosed with COVID-19, confirmed by a molecular test and a commercial quantitative ELISA, and serum from a healthy, non-infected ferret obtained prior to pandemic COVID-19 situation as negative control. The same positive and negative sera were used for all assays. All samples were run in duplicate. The cutoff was set to 0.250 Optical Density units (OD units) (mean + 3 standard deviations of values from 61 ferrets obtained prior the COVID-19 situation in 2020) and the results above this value were considered positive.

Anticoagulated blood samples were analysed by an automated hematology analyser (LaserCyte Idexx, Westbrook, ME, USA) to perform a complete cells blood count. Clinical biochemistries were analysed with an automatic analyser (Catalyst One Idexx, West-brook, ME, USA) including the following parameters: alanine amino-transferase (ALT), alkaline phosphatase (ALKP), glucose (GLU), total protein concentrations (TP), albumin (ALB), globulins (GLOB), creatinine (CRE) and blood urea nitrogen (BUN).

## 3. Results

### 3.1. Characterization of the Animals under Study

In total, 127 client-owned ferrets were sampled including 72 males and 55 females. The ferrets had a mixture of coat colors and no ferrets were neutered surgically. The age of the ferrets ranged from five months to eight years old. The COVID-19 status of the pet owners was unknown except in two households in which four domestic ferrets (three client-owned ferrets without respiratory symptoms and one ferret with swollen tonsils without any other respiratory symptoms) were co-living with a laboratory-confirmed COVID-19 human patient.

A single sample was obtained from 83 client-owned ferrets, out of the 127, at the time of vaccination or routine annual healthcare examination. The other 44 client-owned ferrets were tested from two to six different times during the study period for different medical reasons, most of them because of a previously diagnosed chronic disease or preventive healthcare check-ups.

### 3.2. Serological Prevalence of SARS-CoV-2 Infection in Client-Owned-Ferrets

The seroprevalence of SARS-CoV-2 infection was 1.57 %. Among the 127 household ferrets, two ferrets were seropositive detected by ELISA, with OD units 0.285 and 0.300. The presence of antibodies against RBD was detected in two males (F 18 and F 31) and both seropositive samples were obtained in June. Both ferrets resulted as seronegative in a previous serum sample obtained in March (F 31) and April (F 18).

### 3.3. Health Condition in SARS-CoV-2 Seropositive Animals and Follow Up

F18 is a three years old intact male ferret and lives together with another ferret (four years old) which was seronegative in a sample obtained in October 2020. Furthermore, F18 was under treatment with toceranib in combination with metronomic cyclo-phosphamide/meloxicam therapy since April 2020 after prepuce and penile amputation for treatment of secretory apocrine adenocarcinoma of the preputial skin. Complete blood cell count and serum biochemical profile results showed hyperglobulinemia (Table S1; Supplementary Materials). In the same way, in a follow-up visit in October 2020, four months since F18 resulted seropositive to SARS-CoV-2, hematology revealed neutrophilia with lymphopenia and monocytosis (Table S1; Supplementary Materials) owing to an acute ulcerative-necro-suppurative cystitis due to Burkholderia stabilis. The level of anti-SARS-CoV-2 antibodies in F18 persisted and was the same in the sample obtained 129 days after the first positive result (October 2020).

Furthermore, F 31 is a five year old intact male ferret which is under continuous medical check-ups owing to a splenomegaly with marked homogenous enlargement of the spleen, a marked enlargement of the mesenteric lymph nodes observed by ultrasound, and a hyperglobulinemia revealed in blood, which all were detected in March 2020 without any other clinicopathological findings (Table S1; Supplementary Materials). Considering SARS-CoV-2 ELISA results, F31 resulted as seronegative in a serum sample obtained 128 days after the ferret was tested positive for SARS-CoV-2.

### 3.4. Health Condition in Animals with Diagnosis of FRECV and FRSCV

One ferret testing positive for FRECV was included in this study (F 7 (RECV1)). This patient, a three year old intact male domestic ferret, showed general clinical signs of lethargy, anorexia, vomiting and had foul-smelling, green mucous diarrhea during two days after contact with a new young ferret at home. Complete blood cell count and bio-chemical profile were unremarkable. Feces from these ferrets resulted positive to FRECV (RNA) by Real Time PCR (RT-PCR).

In the same way, serum samples from two ferrets with FRSCV diagnosis (F 33 (FRSCV1) and F 9 (FRSCV2) were included in the study. FRSCV1, a 16 month old intact female domestic ferret, was presented for a posterior paresis, weight loss, anorexia and lethargy. Biochemistry and hematology revealed hyperproteinemia with hypergammaglobulinemia and anemia. An abdominal ultrasonography examination revealed splenomegaly and hypoechoic abdominal masses with abnormal echogenicity of the abdominal organs. A sample of a mass was collected by an ultrasound-guided fine-needle aspiration and cytology revealed pyogranulomatous inflammation, consisting of macrophages, neutrophils, and lymphocytes compatible with a diagnosis of systemic corona-virosis. The FRSCV diagnosis was determined by the presence of coronavirus antigens within the pyogranulomatous lesions by immunohistochemistry (IHQ). Equally, FRSCV2, a 15 months old intact female domestic ferret, was presented with weight loss, anorexia, lethargy and neurological signs often associated to meningoencephalitis, such as ataxia and altered mental state. A complete blood cell count showed regenerative anemia and biochemical profile revealed hypergammaglobulinemia. RT-PCRs for Distemper virus, *Toxoplasma gondii, Cryptococcus neoformans and Neospora caninum* were negative on cerebrospinal fluid analysis. The ferret was euthanized, and a post-mortem examination with subsequent histopathology findings were consistent with a diagnosis of coronavirus pyogranulomatous meningitis with positive result to the presence of FRSCV by IHQ. Serum samples from these ferrets (FRECV1, FRSCV1 and FRSCV2) resulted negative for SARS-CoV-2 by ELISA (Table 1).

### 3.5. SARS-CoV-2 in Ferrets with Exposure to Confirmed COVID-19 Infected Humans

SARS-CoV-2 antibodies were detected in serum samples obtained from four ferrets living with positive SARS-CoV-2 humans (F14, F 19, F 22 and F 24) (Table 1).

Three ferrets (F 14, F 19 and F 22) lived with a COVID-19 infected symptomatic human with a positive PCR SARS-CoV-2 result in March. Blood samples from F 22 were obtained one month and two months after the exposure to the confirmed COVID-19 infected human. Equally, blood samples from F 14 were obtained one month and five months after exposure to the infected human. Four blood samples from F 19 were taken one, four, five and six months after contact with the positive owner. In this sense, respiratory symptoms in the ferret were observed a few weeks after a positive PCR result of the COVID infected owner. A respiratory upper tract inflammation with swollen tonsils was detected on physical examination. In a follow-up three months later, the respiratory upper tract inflammation persisted without any improvement of the symptoms. Thus, an oropharyngeal swab was collected from F 19 for PCR analysis including SARS-CoV-2, canine distemper virus and Influenza A virus with a negative result for all pathogens. In the same way, patient F 19 was seronegative to SARS-CoV-2 with OD unit 0.069 at the same time as the negative SARS-CoV-2 PCR result.

A blood sample was obtained from another pet-ferret (F 24) ten days after exposure to an asymptomatic COVID infected child. In the same way, the owners avoided close child-to-ferret contact from the moment they knew the positive result.

Serum samples from these ferrets tested negative for SARS-CoV-2 by ELISA (Table 1).

## 4. Discussion

In the public health battle against SARS-CoV-2 questions have been raised about the susceptibility to SARS-CoV-2 of household pet-animals under natural conditions. Sporadic detection of natural SARS-CoV-2 cases in animals have occurred alongside successful experimental infections of pets (such as cats, dogs and ferrets), although there is no evidence that infected pets can transmit the virus back to human.

Ferrets (*Mustela putorius furo*) are commonly used as an animal model for viral respiratory infections in humans [7]. Although infections with ferret coronavirus (FRECV and FRSCV) are not considered to be respiratory [3], several studies have shown that ferrets are a suitable animal model to study the pathogenesis of SARS-CoV2 [6,7,8,9]. These studies have demonstrated that the virus is successfully transmitted to co-housed ferrets (direct contact) and via airborne (indirect contacts) [6,10]. In addition, ferrets experimentally infected show similarities to human clinical illness [6,7,11].

Ferrets and minks (*Neovison vison*) are included as members of the same family Mustelidae. Reports showed that humans can become a source of infection for minks, which has resulted in a disease outbreak [12,13]. Equally, epidemiologic evidence supported by genomic sequencing corroborated mink-to-human transmission events in farms [14].

Because ferrets are closely related to minks and there is evidence of mink-to-human and human-to-mink transmission, it is necessary to evaluate the COVID-19 risk represented by household pet-ferrets through epidemiological studies of seroprevalence of SARS-CoV-2 in these animals.

SARS-CoV-2 exhibits a broad tropism for mammalian receptor angiotensin-converting enzyme 2 (ACE2) [15] and this receptor appears to be preferentially used by both SARS-CoV and SARS-CoV-2 to initiate viral entry. However, the domestic ferret ACE2 sequences displayed an 82.6 % overall identity compared to human ACE2 [3]. If SARS-CoV-2 uses the ACE2 receptor to infect non-human hosts, little is known about the reason for the susceptibility of ferrets and other animals, including dogs and cats, to SARS-CoV-2, but it is likely to be due to the similarities in ACE2 [3].

The RBD of the Spike protein of SARS-CoV-2 allows the virus to enter host cells through the human ACE2 and it is used as an antigen for specific antibody detection [16]. Diagnostic results obtained among ELISA tests influenced by the type of antigen demonstrated, RBD or S antigens, provide better diagnostic results [17,18]. Moreover, the RBD antigen possesses low amino acid sequence identity among coronavirus species, preventing possible cross-reactivity between RBD protein of SARS-CoV-2 and RBD protein of FRECV and FRSCV. Nonetheless, three ferrets with ferret coronavirus diagnosis included in the study (one ferret tested FRECV positive by RT-PCR and two ferrets with a FRSCV diagnosis determined by IHQ) resulted as seronegative to SARS-CoV-2, therefore discarding possible false positive results.

This study revealed SARS-CoV-2 antibodies in two household pet-ferrets (1.57%) using the ELISA technique from a total of 194 blood samples obtained from 127 client-owned-ferrets. According to the owners, no apparent clinical signs similar to SARS-CoV-2 symptoms in humans were observed in any of two seropositive ferrets during the year of the study. In this sense, no human living with the pet-ferrets presented symptoms related to SARS-CoV-2 during the study period.

It is hypothesized that SARS-CoV-2 might be transmitted via airborne and closed environments contributing to the secondary transmission of the virus, thereby promoting the super-spreading phenomenon [5]. Therefore, the possible source of infection is probably linked to the exposure of the household ferrets to asymptomatic SARS-CoV-2 infected humans, these circumstances also being described for stray cats [12], tigers and lions living in zoos [19]. However, serum samples from four ferrets with documented exposure to confirmed COVID-19 infected humans resulted negative to SARS-CoV-2, perhaps because COVID-19 positive ferret owners tried to avoid contact with their pets to prevent possible complications, instead of what happens with COVID-19 asymptomatic pet owners. By contrast, a recent survey of household cats and dogs of laboratory-confirmed COVID-19 patients found that seropositivity was significantly greater among pets from COVID-19 positive households compared to those with owners of unknown status [20].

SARS-CoV-2 effects on animal health are almost unknown. In humans, the severity of the disease is determined, in most cases, by patient’s comorbidity status [21]. However, in domestic ferrets the disease pathophysiology and predisposing factors are unknown. In this study, no evidence of clinical signs potentially caused by SARS-CoV-2 was attributed to F18 and F31. Complete blood cell counts revealed hematological values within normal limits and serum biochemistry profiles showed elevated globulins in both patients (Table S1; Supplementary Materials). Hyperglobulinemia is found in many types of inflammation, determinate infections or certain neoplasms, but this finding was detected previously by the SARS-CoV-2 seropositivity in both patients. F18 at the moment of SARS-CoV-2 antibodies detection was under chemotherapy which could induced immunosuppression in the patient and might have acted as a promoting factor for the presence of SARS-CoV-2 infection. F31 was under continuous check-ups because a splenomegaly and marked enlargement of the mesenteric lymph nodes detected months before SARS-CoV-2 antibodies were detected in the patient. Splenic enlargement is a very common and nonspecific finding in adult ferrets and the causes are multiple, including extramedullary hematopoiesis, lymphosarcoma and other neoplasms such as hemangiosarcoma, cardiomyopathy or chronic infections [22]. Equally, lymph nodes enlargement in ferrets is associated with chronic inflammation or chronic infections. In ferret F31, the cause of splenic and mesenteric lymph nodes could not be determined but probably were related to a chronic infection, inflammation or other concomitant debilitating diseases, so these conditions may act as risk factors for SARS-CoV-2 in ferrets, and therefore should be investigated.

Both seropositive samples were obtained in June, at the end of the first state of alarm aiming to control the spread of the coronavirus pandemic declared in the country by the Spanish Government. That lockdown remained for three months in which pet-owners stayed at home more than habitually, therefore increasing close contact with their pets.

Other preprint epidemiological survey of SARS-CoV-2 in dogs and cats from households in Italy evidence that 3.4% of dogs and 3.9% of cats had measurable SARS-CoV-2 antibody titers [23], detecting higher incidence than the one detected in household ferrets in this study.

Antibodies play an important role in neutralizing virus and provide protection to the host against viral re-infection [24]. Currently, our understanding of antibody responses following infection with SARS-CoV-2 is limited and different results have been reported [25]. The concentration of anti-RBD IgG antibodies persisted at detectable levels in human patients beyond 90 [26] or 120 [27] days after symptom onset, and seroconversion was only observed in a small percentage of individuals according to different studies [28]. More recently, it was found that most mild-moderate COVID-19 patients generated antibodies against Spike protein that presented neutralizing activity, which could be detected five months after disease onset [28]. However, these findings differ from other studies suggesting a more rapid waning in anti-RBD titters following mild or asymptomatic human SARS-CoV-2 infection [29,30]. In this study, anti-RBD SARS-CoV-2 antibodies persisted at least for 129 days in one seropositive asymptomatic ferret F18. In contrast, F31 resulted as seronegative after 128 days.

Determination of the susceptibility of various animal species to infection with SARS-CoV-2 and the role of ferrets and other companion animals in the epidemiology of the disease will be critical in informing appropriate human and veterinary public health responses to this pandemic [31].

Because recent reports showed SARS-CoV-2 infection and COVID-19 symptoms in minks and ferrets as animal models, an abundance of caution is needed and the epidemiological role of this virus in client-owned ferrets has to be investigated, being necessary in order to confirm their susceptibility through future laboratory testing studies. Due to the possibility of transmission of the SARS-CoV-2 virus to ferrets, owners should take strict precautions to avoid the infection, this situation being also recommended for other pets and wild mammals [31,32].

## 5. Conclusions

This study reports for the first time the evidence of household pet ferrets’ exposure to SARS-CoV-2 in Spain to date. The presence of anti-RBD SARS-CoV-2 antibodies persisted at detectable levels in a seropositive SARS-CoV-2 domestic ferret beyond 129 days since the first-time antibodies were detected. In this sense, serological assays represent a feasible option to elucidate the host range of SARS-CoV-2 and the prevalence in susceptible species including ferrets.

## Figures and Tables

**Table 1:** Serological evidence in animals with follow-up (the cutoff was set to 0.250 Optical Density units).

Ferret Number	Dates													
	Gender	Age (y)	Health Condition	January	February	March	April	May	June	July	August	September	October	November
F 1	♀	8	ED	0.059	0.057	na	na	na	na	na	na	na	na	na
F 2	♀	2	UD	0.050	na	0.057	na	na	na	na	na	na	na	na
F 3	♂	7	CRD/CD	0.066	na	na	0.046	na	na	na	na	na	na	0.049
F 4	♂	5	UD	0.064	na	na	na	na	na	na	na	0.078	na	na
F 5	♂	5	CRD	0.063	na	0.091	na	na	na	na	na	na	0.069	na
F 6	♂	5	N	0.061	na	na	0.085	na	na	na	na	na	na	na
F 7 (FRECV1)			GD	na	0.076	0.049	na	na	na	na	0.083	na	na	na
F 8	♀	4	Leish	na	0.088	0.104	0.074	na	na	na	na	na	na	na
F 9 (FRSCV2)	♀	1	FRSCV	0.068	0.069	na	na	na	na	na	na	na	na	na
F 10	♂	5	ED	na	0.098	na	na	na	0.130	0.100	0.121	na	na	na
F 11	♂	5	S	na	0.075	na	na	na	0.069	0.081	na	na	na	na
F 12	♀	5	HD	na	na	0,064	na	na	0.118	na	na	na	na	na
F 13	♀	5	UD	na	na	0.107	na	na	na	0.097	na	na	na	na
F 14	♂	3	GD	na	na	na *	0.091 *	na	na	na	0.078 *	na	na	na
F 15	♀	6	Leish	na	na	0.076	0.123	0.107	na	na	na	na	na	na
F 16	♂	1	NAD	na	na	0.059	na	na	0.109	na	na	na	na	na
F 17	♂	1	NAD	na	na	0.071	na	0.069	na	na	na	na	na	na
F 18	♂	3	N	na	na	na	0.079	na	0.285	na	na	na	0.285	na
F 19	♂	4	CRD	na	na	na *	0.068 *	na	na	0.069 †*	0.085 *	0.048 *	na	na
F 20	♀	4	HD	na	na	na	0.041	na	na	na	na	na	0.049	na
F 21	♂	<1	NAD	na	na	na	na	na	na	na	na	na	0.071	0.071
F 22	♂	4	NAD	na	na	na *	0.102 *	0.092 *	na	na	na	na	na	na
F 23	♀	8	UD	na	na	na	0.082	0.097	na	na	na	na	na	na
F 24	♀	3	GD/N	na	0.056	0.066	0.068	0.110	na	na	na	na	na	0.042 *
F 25	♂	4	S	na	na	na	na	0.076	0.101	na	na	na	na	na
F 26	♀	3	NAD	na	na	na	na	0.073	0.153	na	na	na	na	na
F 27	♀	4	Leish	na	na	na	0.048	na	0.092	0.069	0.066	0.066	0.042	na
F 28	♂	6	ED	na	na	na	na	na	0.064	na	0.066	na	na	na
F 29	♂	4	GD	na	na	na	na	na	0.068	0.074	na	na	na	na
F 30	♀	3	GD	na	na	na	na	na	0.058	na	0.067	na	na	na
F 31	♂	5	S	na	na	0.071	na	na	0.300	na	na	na	0.059	na
F 32	♀	5	ED	na	na	na	na	na	0.087	na	0.081	na	na	0.112
F 33 (FRSCV1)	♀	1	SC	na	na	na	na	na	0.054	na	na	na	0.085	0.046
F 34	♂	4	GD	na	na	na	na	na	0.058	na	0.067	na	na	na
F 35	♂	6	CD	na	na	na	na	na	0.073	na	na	na	na	0.055
F 36	♀	5	NAD	na	na	na	na	na	0.085	0.074	na	na	na	na
F 37	♀	1	NAD	na	na	na	na	na	0.068	0.061	na	na	na	na
F 38	♀	4	N	na	na	na	na	na	na	0.071	0.075	na	na	na
F 39	♂	4	HD	na	na	na	na	na	na	0.112	0.082	0.115	na	0.091
F 40	♂	2	UD	na	na	na	na	na	na	0.070	0.075	na	na	na
F 41	♀	8	ED	na	na	na	na	na	na	0.063	0.070	na	na	na
F 42	♂	4	S	na	na	na	na	na	na	0.068	na	na	0.066	na
F 43	♀	3	NAD	na	na	na	na	na	na	na	na	na	0.057	0.057
F 44	♂	5	NAD	na	na	0.092	na	na	na	na	na	na	na	0.072

Abbreviations: CD cardiovascular disease; CRD chronic respiratory disease; ECE enteric coronavirus; ED endocrine disease; GD gastrointestinal disease; HD hepatic disease; Leish Leishmaniosis; UD urinary disease; N neoplasia; NAD nothing abnormal detected, na not available; S splenomegaly and/or enlargement lymph nodes; SC systemic coronavirus; * Samples from ferrets obtained after confirmation of COVID-19 infected humans; † Negative SARS-CoV-2 PCR; (F18) & (F31) SARS-CoV-2 seropositive ferrets; (FRECV1) Positive PCR enteric coronavirus ferret; (FRSCV1 & FRSCV2) Positive systemic enteric coronavirus ferrets.

## Data Availability

The data presented in this study are available on request from the corresponding author. The data are not publicly available due to privacy restrictions.

## Supplementary Materials

The following are available online at https://www.mdpi.com/2076-2615/11/3/667/s1, Table S1: Body weight, hematological and biochemical parameters determined to seropositive SARS-CoV-2 ferrets.
