# Supplementary material for: SARS-CoV-2 Seroprevalence in Household Domestic Ferrets (Mustela putorius furo)
Source: Animals (Basel). 2021 Mar 2;11(3):667. doi: 10.3390/ani11030667 (PMC8001492; doi:10.3390/ani11030667)
Supplement: Supplementary file 1 [file animals-11-00667-s001.pdf]

Supplementary Materials

Table S1. Body weight, hematological and biochemical parameters determined to seropositive Scheme 2. ferrets (F 18 and F 31) in the medical check-ups before tested positive (a), the month tested positive (b) (F 18 and F 31) and during follow-ups (c) (F 18 and F 31) and (d) (F 18).

**Parameter**	**F 18 April (a)**	**F 18 June (b)**	**F 18 August (c)**	**F 18 October (d)**	**F 31 March (a)**	**F 31 June (b)**	**F 31 October (c)**	**Reference Range**
Body weight (g)	1380	1430	1400	1315	1205	1180	1305	900-1500
Haematology								
WBC (K/µL)	9.76	5.21	4.29	8.72	5.00	4.90	4.84	2-10
Neutrophils (K/µL)	**7.15**	2.88	2.52	**5.99**	2.02	2.60	2.40	0.62-3.30
Lymphocytes (K/µL)	1.59	1.65	1.20	**0.97**	1,02	1.37	1.80	1-8
Monocites (K/µL)	0.78	0.46	0.41	**1.43**	0.55	0.78	0.52	0.18-0.90
Eosinophils (K/µL)	0.22	0.21	0.15	0.30	0.37	0.12	0.10	0.10-0.60
Basophils (K/µL)	0.03	0.01	0.01	0.03	0.05	0.03	0.03	0.00-0.10
RBC (M/µL)	10.07	8.14	**12.01**	10.62	8.59	8.05	10.16	6.35-11.20
Haematocrit (%)	50.7	39.7	**55.1**	52.3	48.9	46.4	51.3	37.0-55.0
Haemoglobin (g/dL)	**17.7**	16.5	**19.3**	16.6	16.9	16.0	17.0	11.0-17.0
MCV (fL)	50.4	48.8	45.9	49.2	51.05	54.0	50.5	45.0-55.0
MCH (pg)	17.3	17.3	16.1	15.6	17.2	18.0	18.0	14.0-18.0
MCHC (g/dL)	34.9	23.8	35.01	33.10	34.6	34.5	35.0	32.0-35.0
RDW (%)	19.1	19	**16.3**	**16**	19.0	19.0	19.2	19.0-25.0
Platelets (K/µL)	650	567	525	501	326	344	300	270-880
Blood Chemistry								
ALT (U/L)	83	82	85	83	85	51	84	82-289
ALKP (U/L)	11	18	17	19	28	18	11	9-84
Glu (mg/dL)	105	128	110	107	104	94	**79**	94-207
Crea (mg/dL)	0.9	0.4	**0.3**	0.4	0.9	0.4	0.6	0.4-0.9
BUN (mg/dL)	28	45	21	36	23	27	34	10-45
Total protein (g/dL)	6.4	6.5	7.0	6.1	6.7	6.8	7.6	5.2-7.3
Albumin (g/dL)	2.7	2.6	3.2	2.8	3.2	2.9	0.4	2.6-3.8
Globulins (g/dL)	**3.7**	**3.8**	**3.8**	**3.2**	**3.5**	**3.9**	**4.2**	1.8-3.1
ELISA serology (OD)	0,079	**0.285**	nd	**0.285**	nd	**0.300**	0.059	Cutt-off: ≥0.250

Abbreviations: ALB Albumin, ALT Alanine amino-transferase, ALKP Alkaline Phosphatase, BUN Blood Urea Nitrogen, Crea Creatinine ELISA Enzyme-Linked Immunosorbent Assay, GLOB Globulins, Glu Glucose, MCH Mean Corpuscular Haemoglobin, MCHC Mean Corpuscular Haemoglobin Concentration, MCV Mean Corpuscular Volume, RBC Red Blood Count, RDW Red Blood Cell Distribution Width, TP Total Protein concentrations, WBC White Blood Count, nd not determined. Abnormalities are highlighted in bold.
